# P-1419. Comparison of Respiratory Syncytial Virus-Specific Antibody Durability in Pregnant/Postpartum Individuals and Older Adults After RSV Vaccination

**DOI:** 10.1093/ofid/ofaf695.1606

**Published:** 2026-01-11

**Authors:** Alisa B Kachikis, Collrane J Frivold, Mindy Pike, Jennifer E Stolarczuk, Kittredge McVey, Erica Clark, Sandra McAteer, Ioana-Maria Saidac, Alex Harteloo, Marco Carone, Grace Marshall, Linda O Eckert, Janet A Englund, Helen Y Chu

**Affiliations:** University of Washington Department of Obstetrics & Gynecology, Seattle, WA; University of Washington, Seattle, Washington; University of Washington, Seattle, Washington; University of Washington, Seattle, Washington; University of Washington, Seattle, Washington; University of Washington, Seattle, Washington; University of Washington, Seattle, Washington; University of Washington, Seattle, Washington; University of Washington, Seattle, Washington; University of Washington, Seattle, Washington; University of Washington, Seattle, Washington; University of Washington, Seattle, Washington; Seattle Children’s Hospital/Univ. Washington, Seattle, Washington; University of Washington, Seattle, Washington

## Abstract

**Background:**

Respiratory syncytial virus (RSV) vaccination is recommended in the third trimester of pregnancy to prevent infant illness. Little data exists regarding RSV-specific antibody (Ab) durability following vaccination during pregnancy to inform need for revaccination in subsequent pregnancies; however, data from older adult RSV vaccines suggests effectiveness out to 2-3 years. We compared durability of RSV-specific Ab levels at 12-15 months following RSV vaccination between pregnant/postpartum individuals and older adults.

**Figure:**
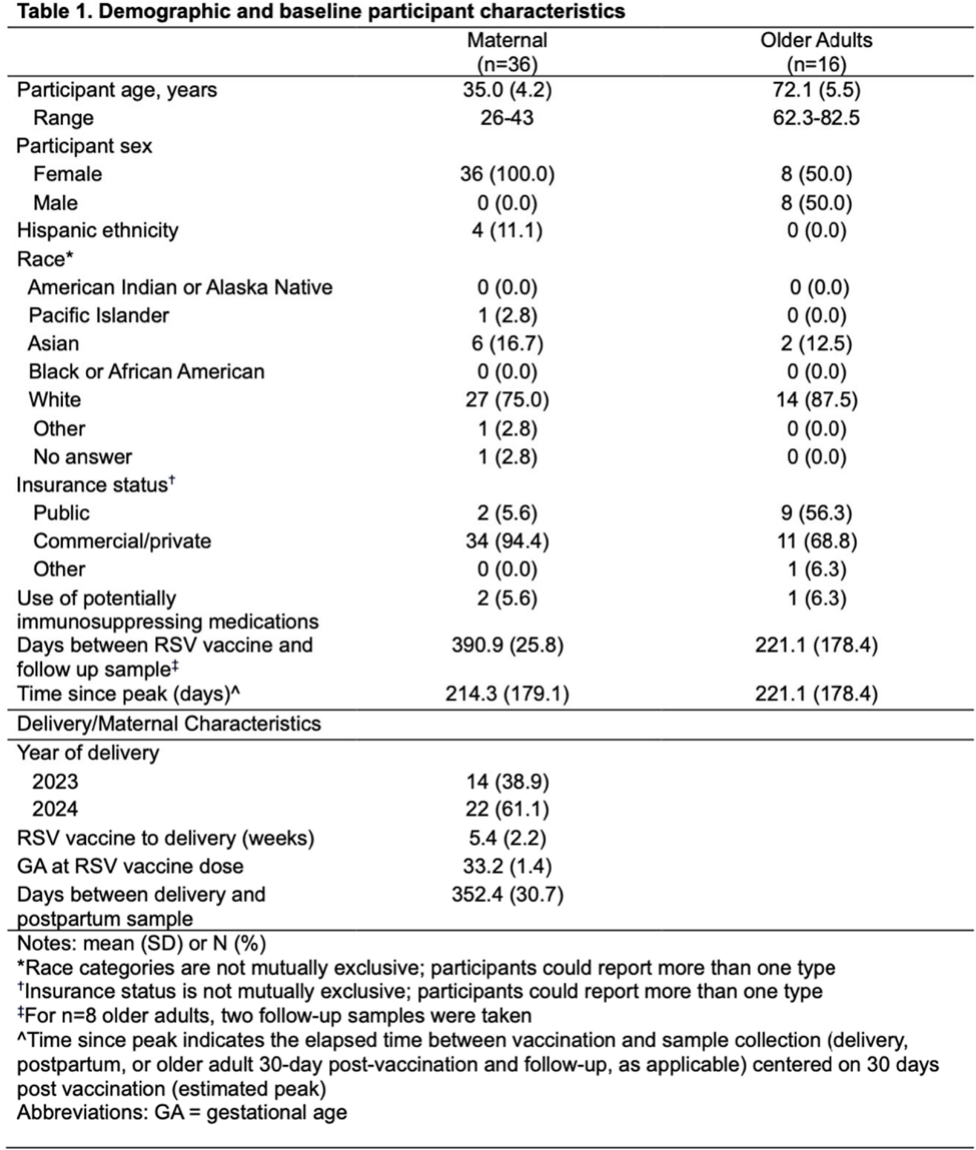

**Figure:**
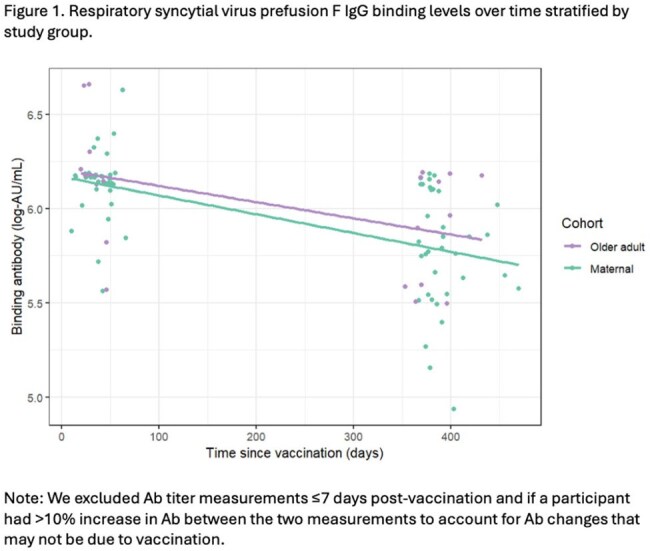

**Methods:**

We conducted a prospective cohort study among pregnant individuals who received an RSV vaccination during pregnancy, and among older adults after their first RSV vaccine. We tested maternal samples at delivery, older adult samples at one month post-vaccination, and both populations at 12-15 months following vaccination for RSV pre-F binding Ab (Mesoscale Diagnostics). To characterize and compare the change in RSV Ab levels over time, we fit a linear model with generalized estimating equations.

**Figure:**
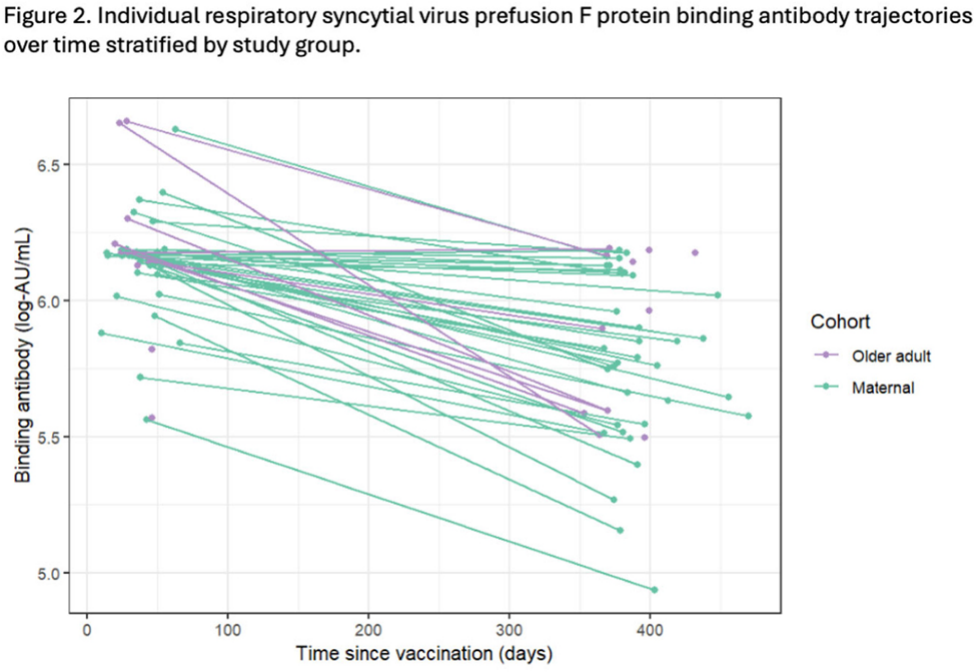

**Figure:**
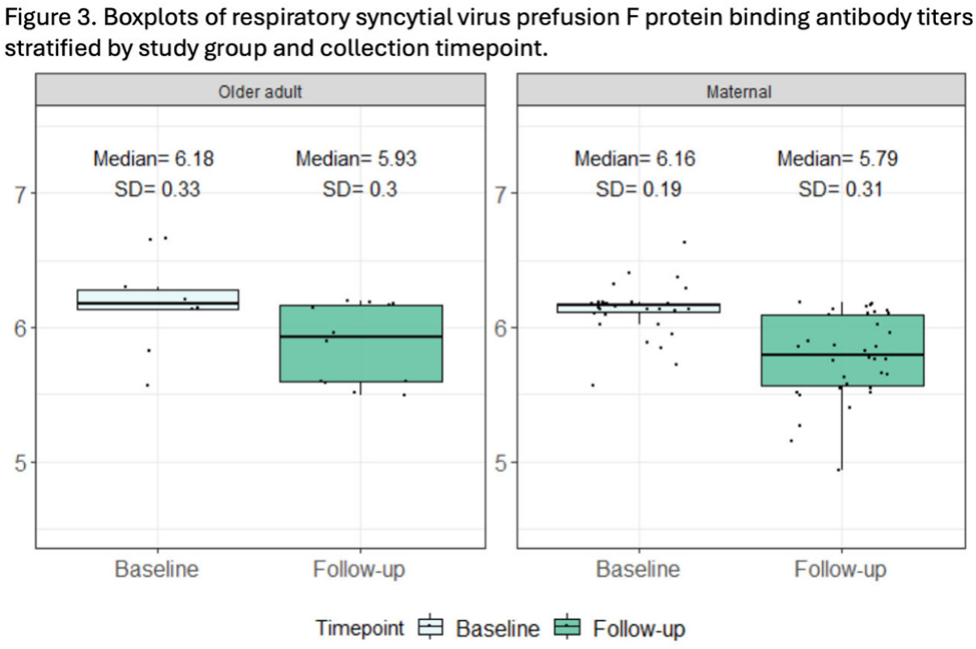

**Results:**

We tested samples following RSV preF vaccine for RSV preF IgG in 36 individuals vaccinated during pregnancy (mean age, 35.0 [±4.2] years) and in 16 older adults (mean age, 72.1 [±5.5] years). Among maternal and older adult participants, median log-binding antibody titers were 6.16 log-AU/mL and 6.18 log-AU/mL, respectively, at baseline compared to 5.79 log-AU/mL and 5.98 log-AU/mL, respectively, at follow-up. We estimated that RSV preF IgG half-life was 696 (95% CI: 550; 842) and 806 (95% CI: 111; 1,501) days in the maternal and older adult groups, respectively. We found the patterns of change in mean post-vaccination Ab levels over time were similar between the maternal and older adult subgroups (p-value=0.73).

**Conclusion:**

Our results show that maternal Ab titers waned at similar rates compared to those of older adults. We encourage further studies with large diverse cohorts to assess Ab durability following maternal RSV vaccination. Revaccination against RSV in subsequent pregnancies may be warranted to address postpartum Ab waning for optimal prevention of infant illness, though the optimal window is not yet defined.

**Disclosures:**

Alisa B. Kachikis, MD, MSc, Pfizer: Advisor/Consultant|Pfizer: Grant/Research Support|SCL Seqirus: Honoraria Janet A. Englund, MD, AstraZeneca: Board Member|AstraZeneca: Grant/Research Support|Cidarra: Member Data Safety Monitoring Board|GlaxoSmithKline: Advisor/Consultant|GlaxoSmithKline: Grant/Research Support|Meissa Vaccines: Advisor/Consultant|Merck: Advisor/Consultant|Merck: Grant/Research Support|Moderna: Advisor/Consultant|Moderna: Grant/Research Support|Pfizer: Advisor/Consultant|Pfizer: Grant/Research Support|Shionogi: Grant/Research Support Helen Y. Chu, MD, MPH, Roche: Advisor/Consultant|Vir: Advisor/Consultant

